# Validation of the Six-item Female Sexual Function Index in Middle-Aged Brazilian Women

**DOI:** 10.1055/s-0039-1692694

**Published:** 2019-07-09

**Authors:** Mona Lúcia Dall'Agno, Charles Francisco Ferreira, Fernanda Vargas Ferreira, Faustino R. Pérez-López, Maria Celeste Osório Wender

**Affiliations:** 1 Universidade Federal do Rio Grande do Sul, Porto Alegre, RS, Brazil; 2 Department of Obstetrics and Gynecology, Lozano-Blesa University Hospital, Zaragoza, Spain

**Keywords:** climacteric, cross cultural comparison, menopause, test reliability, female sexual health, climatério, comparação transcultural, menopausa, reprodutibilidade dos testes, saúde sexual da mulher

## Abstract

**Objective** To validate the six-item female sexual function index (FSFI-6) in middle-aged Brazilian women.

**Methods** Cross-sectional observational study, involving 737 (premenopausal *n* = 117, perimenopausal *n* = 249, postmenopausal *n* = 371) Brazilian sexually active women, aged between 40 and 55 years, not using hormonal contraceptive methods. The Brazilian FSFI-6 was developed from the translation and cultural adaptation of the Portuguese FSFI-6 version. The participants completed a general questionnaire, the FSFI-6, and the menopause rating scale (MRS). The validation was performed by AMOS 16.0 software (SPSS, Inc., Chicago, IL, USA) for a confirmatory factor analysis (CFA). The chi-square of degrees of freedom (χ2/df), the comparative fit index (CFI), the Tucker-Lewis index (TLI) and the root-mean-square error of approximation (RMSEA) were used as indices of goodness of fit. Cronbach α coefficient was used for internal consistency.

**Results** The process of cultural adaptation has not altered the Brazilian FSFI-6, as compared with the original content. The CFA for the FSFI-6 score showed an acceptable fit (χ2/df = 3.434, CFI = 0.990, TLI = 0.980, RMSEA = 0.058, 90% confidence interval (90%CI) = 0.033–0.083, *p* ≤ 0.001) and a good reliability was established in FSFI-6 and MRS (Cronbach α = 0.840 and = 0.854, respectively). In addition, 53.5% of the sample had low sexual function.

**Conclusion** The FSFI-6 was translated and adapted to the Brazilian culture and is a consistent and reliable tool for female sexual dysfunction screening in Brazilian middle-aged women.

## Abstract (pt)

**Resumo:**

**Objetivo** Validar o Índice de Função Sexual Feminina - 6 itens (FSFI-6, na sigla em inglês) para mulheres brasileiras de meia-idade.

**Métodos** Estudo transversal observacional que incluiu 737 (pré-menopausa *n* = 117, perimenopausa *n* = 249, pós-menopausa *n* = 371) mulheres brasileiras sexualmente ativas, entre 40 e 55 anos, sem métodos contraceptivos hormonais. A versão brasileira do FSFI-6 foi desenvolvida através da tradução e adaptação cultural da versão portuguesa do questionário. As participantes preencheram um questionário com dados gerais, o FSFI-6 e a escala de avaliação da menopausa (*menopause rating scale* [MRS]). A validação do instrumento se deu através de análise fatorial confirmatória (CFA, na sigla em inglês), realizada pelo software AMOS 16.0 (SPSS, Inc., Chicago, IL, EUA). Qui-quadrado sobre graus de liberdade (χ2/df), índice de ajuste comparativo (CFI, na sigla em inglês), índice de Tucker-Lewis (TLI) e raiz média dos quadrados dos erros de aproximação (RMSEA, na sigla em inglês) foram utilizados como índices de adequação de ajustes. O coeficiente alfa de Cronbach foi utilizado para avaliar a consistência interna.

**Resultados** O processo de adaptação cultural não alterou a versão brasileira do FSFI-6, comparado ao conteúdo original. O CFA para o escore do FSFI-6 demonstrou ajuste aceitável (χ2/df = 3,434; CFI = 0,990; TLI = 0,980; RMSEA = 0,058; 90% IC = 0,033 a 0,083; *p* ≤ 0,001). Demonstrou-se boa confiabilidade entre FSFI-6 e MRS (alfa de Cronbach = 0,840 e = 0,854, respectivamente). Do total, 53,5% da amostra apresentou baixa função sexual.

**Conclusão** O FSFI-6 foi traduzido e adaptado culturalmente, e é uma ferramenta consistente e confiável no rastreamento de disfunções sexuais em mulheres brasileiras de meia-idade.

## Introduction

Sexual function (SF) is an important component of the quality of life (QoL) of peri- and postmenopausal women.^1^ The climacteric population is progressively growing all over the world and is characterized by a range of signs and symptoms, with low sexual desire being a well reported one.^2^ ^3^ ^4^ Due to estrogen deficiency, menopause is related to other consequences in SF, as vaginal atrophy, reduction in lubrication and dyspareunia.^5^

The climacteric period impacts sexuality through the interaction between hormonal, biological, social, cultural and other individual characteristics, and can negatively impact the QoL.^6^ ^7^ Although female sexual dysfunction (FSD) seems to increase with age,^5^ its actual prevalence is variable according to the literature.^8^ Cultural factors, physician-patient relationship, and lack of standardization of diagnosis are possibly determinants of this variation.^8^

Due to its complexity,^9^ questionnaires to assess female sexual health have been developed, providing a better understanding of subjective and objective aspects, allowing comparisons between individuals and populations.^10^ In addition, they are inexpensive, non-invasive, useful for health professionals and generally, self-fulfilling.

One of the most worldwide used instruments for assessment of FSD is the female sexual function index (FSFI),^11^ ^12^ with reliability/validity demonstrated extensively in several studies for different populations.^8^

The original FSFI has 19 items and includes the 6 domains of FSD,^11^ according to the International Classification of Diseases-10 (ICD-10) and the Diagnostic and Statistical Manual of Mental Disorders (DSM-IV): desire, arousal, lubrication, orgasm, satisfaction, and pain.^11^ The FSFI was validated for many languages and populations, including some Brazilian versions that have already been published.^12^ ^13^ ^14^ ^15^ ^16^

In an attempt to obtain a smaller and faster instrument, a short version of the FSFI was proposed, consisting of six items.^17^ The selected items refer to the six domains of the FSD (one item for each domain), maintaining the psychometric properties and reliability of the original tool.^17^ This shorter version was validated in Spanish,^18^ ^19^ ^20^ ^21^ Korean,^22^ and Portuguese for a sample of Portuguese women.^23^

Despite the importance of the SF for the QoL, it is rarely investigated in the women's health care.^6^ ^11^ ^24^ The minority of women with a sexual problem seek for professional help.^24^ The use of an easier and faster questionnaire may be a relevant tool in the medical assistance of these women, thus allowing adequate assessment of SF.

Therefore, this research aimed to validate the FSFI-6, assessing middle-aged women's sexual function and associated variables in the Southern region of Brazil. It is suggested that the validation results in a reliable instrument applicable in research and clinical practice, as well as in the publication of important data on the female sexual health.

## Methods

### Study Design and Participants

This is a cross-sectional study, performed in areas of free access and transit of the population (e.g., parks, squares, streets, shopping) in the Southern region of Brazil, from January to October 2017, involving middle-aged women. The cities where the questionnaires were applied are located in the three states of the Southern region of Brazil, with populations varying between 47,000 and 1,484,000 inhabitants. Ethical approval was obtained from the Institutional Review Board of the Hospital de Clínicas de Porto Alegre (HCPA, Ref. N°. 16–0621). The study involved women in the community, aged between 40 and 55 years, classified as pre-, peri-, or postmenopausal, according to the Stages of Reproductive Aging Workshop +10 (STRAW +10),^3^ not using hormonal contraceptive methods (e.g., contraceptive use of levonorgestrel intrauterine device, combined hormonal contraceptives, progestin only pills or implants), who reported sexual activity in the past 4 weeks and that agreed to participate. Women unable to understand the survey or having incapacity imposing difficulties during the filling the questionnaire were excluded. After being informed of the study (e.g., objectives and tools used) and providing written consent, the surveyed women were requested to voluntarily fill out a general questionnaire containing health, habits and sociodemographic, data, the FSFI-6 and the menopause rating scale (MRS), with the help of a drawing board. Sample size calculation was performed using the software WinPEPI, PEPI-for-Windows, version 11.65 based on the fact that 30 to 50% of middle-aged women would present lower sexual function (Blümel et al,^5^; Pérez-López et al^19^; Llaneza et al^25^). Hence, a minimal sample of 715 participants was calculated, considering a 5% of losses, 5% desired precision, and a 99% confidence limit.

### General Questionnaire

A questionnaire was developed by the researchers, and it contains female data, including age (years), partner status (yes/no), marital status, educational level (total years), sexual status in the past 4 weeks (active or inactive), parity, professional status, number of people living in the household, and family income (in minimum wages). Health data related to menopausal status (pre-, peri-, and postmenopausal), according to the STRAW + 10^3^ were collected, such as menopause age, type of menopause (natural or surgical), pharmacological treatment for menopausal symptoms (yes/no, type). General health and disease data, surgical procedures performed, psychological problems (depression, anxiety), urinary loss, anthropometric data (weight, height, and body mass index [BMI]), as well as life habits (smoke, alcohol and coffee consumption, physical activity) were assessed.

### The FSFI-6

This instrument is composed of 6 questions based on existing items of the FSFI, each covering one of the original domains: desire, arousal, lubrication, orgasm, satisfaction, and pain.^17^ Each item can provide a score varying from 0 to 5, whose sum provides a total FSFI-6 score.^17^ This is a screening tool aiming to identify women at high risk of FSD.^17^

### The MRS

The MRS scale is a valuable tool for assessing climacteric symptoms and health-related QoL of climacteric women through 11 items distributed in 3 domains: somatic, psychological, and urogenital symptoms.^26^ The score for each question ranges from 0 (absence of symptoms) to 4 (very severe symptoms). The total score is obtained by adding the score of each domain. The higher the score reached, the worse the QoL related to the climacteric symptoms.^27^ ^28^ In addition, symptom severity can be categorized for each domain: absent or occasional (0–4 points), mild (5–8 points), moderate (9–15 points) or severe (≥ 16 points) symptoms.^26^ The Brazilian version of the MRS was already validated.^29^

### Translation and validation

Considering that Brazilian and Portuguese women do not share the exact same cultural and ethnical background, and therefore differences could exist in the understanding and the interpretation of the instrument questions, two stages were used for conducting this study. After obtaining the authorization of the author who validated the FSFI-6 to Portuguese language,^23^ the first stage was performed and included translation and cultural adaptation of the original scale to our context. The instructions of the World Health Organization (WHO) were followed (e.g., translated by two independent and experienced native speakers of the target language translators; reconciliation by the researchers and the translators, who resolves the discrepancies of the forward translations into a single version; back translation by a different bilingual translator; and harmonization between the translations).^30^ The cognitive debriefing was performed with 30 subjects from the study population (community women between 40 and 55 years, 10 each menopausal status) to assess the degree of understanding and testing for cognitive equivalence. At the end of the tool, the question “*Is there a word that has not been understood?*” detects words not understood by women in the early stages of cultural adaptation and validation. These subjects were not included in the analyzes of this study. Review of cognitive debriefing results for finalization and proofreading by the researchers and an expert committee (e.g., three gynecologists, one psychologist, and one biologist) was also performed.^30^ Cultural equivalence was established according to the criteria by Guillemin et al:^31^ at least 85% of the subjects should not show any kind of difficulty to answer each question (e.g., no question should be considered incomprehensible by over 15% of the participants). The second stage was the instrument validation, corresponding to the statistical analysis of its psychometric properties. The validity occurred by the comparison with a specific reference tool (MRS), which has been previously validated for the Brazilian Portuguese.^29^ The final Brazilian FSFI-6 questionnaire is shown in Supplemental Material 1.

## Statistical Analysis

Regarding the data processing, database double entry, review and analysis were performed using the SPSS, version 18.0. (SPSS Inc., Chicago, IL, USA). Symmetric data was expressed as mean and standard error of mean (SEM), or by median and 25^th^ to 75^th^ percentiles (P25–P75). The Shapiro-Wilk test was used to determine the normality of data distribution. Categorical variables were described as absolute (n) and relative (n%) frequencies. According to this, differences in FSFI-6 domains and total scores were analyzed with the Kruskal-Wallis test (bivariate analysis, Dunn *posthoc*) or the chi-squared test, with adjusted residual analysis for independent samples. To examine the construct validity of our model for mathematical FSFI-6, the factor loadings of the variables in each model was calculated with AMOS 16.0 software (SPSS, Inc., Chicago, IL, USA). A confirmatory factor analysis (CFA) was conducted. The chi-squared of degrees of freedom (χ^2^/df), the comparative fit index (CFI), the Tucker-Lewis index (TLI), and the root-mean-square error of approximation (RMSEA) were used as indices of goodness of fit. The internal consistency (criterion validity) of FSFI-6 and MRS instruments was assessed using Cronbach α coefficients.^32^ Spearman ρ coefficients were estimated for determining the correlations between FSFI-6 total scores and variables, including scores obtained with the MRS.

The level of significance was set at 5% for all analyses.

## Results

In total, 737 women were invited and met inclusion criteria of this study, being classified as pre- (*n* = 117), peri- (*n* = 249), or postmenopausal (*n* = 371) according to the STRAW + 10 criteria. They were included and answered a general questionnaire containing health, habits, and sociodemographic data; the Brazilian version of the FSFI-6; and the MRS. The general characteristics of the surveyed women are presented in Table 1. Briefly, most women were aged between 50 and 54 years (39.8%), multiparous (63.9%), married or living with partner (76.1%), currently having a sexual partner (96.3%), and with natural menopause (78.7%). The median (P25–P75) time since menopause onset was 5.00 (3.00–7.50) months, and most of them did not use medication for menopause symptoms (85.6%). Considering health and habits aspects, 116 (15.7%) were smokers, 130 (17.6%) had hypertensive disorder, 40 (5.4%) were diabetic, 193 (26.2%) had a psychiatric condition (e.g., depression symptoms and anxiety) and 116 (15.7%) were sedentary.

**Table 1:** Characteristics of all surveyed women

Female data	*N* = 737
Age (years)	
40–44	117 (15.9)
45–49	210 (28.5)
50–54	293 (39.8)
55–59	117 (15.9)
Parity	
0	78 (10.6)
1	188 (25.5)
≥ 2	471 (63.9)
Educational level (years)	
0–6	109 (14.8)
7–12	316 (42.9)
≥ 13	312 (42.3)
Marital status	
Married or living with partner	561 (76.1)
Divorced	98 (13.3)
Single	65 (8.8)
Widowed	13 (1.8)
Currently has partner	
Yes	710 (96.3)
No	27 (3.7)
Menopausal status	
Premenopausal	117 (15.9)
Perimenopausal	249 (33.8)
Postmenopausal	371 (50.3)
Natural menopause	292 (78.7)
Surgical menopause	79 (21.3)
Time since menopause onset (months)	5.00 [3.00–7.50]
[minimum–maximum]	[0.00–31.00]
Pharmacological treatment for menopause symptoms	
No	631 (85.6)
Systemic hormone therapy	44 (6.0)
Alternative therapies (herbal teas, phytoestrogens)	58 (7.9)
Psychotropics	1 (0.1)
Topic estrogen	3 (0.4)
Habits, lifestyle, health aspects and other issues	
Current smoking	116 (15.7)
Body mass index	26.29 [23.51–30.39]
[minimum–maximum]	[16.00–54.11]
Sedentary lifestyle	116 (15.7)
Hypertension	130 (17.6)
Diabetes	40 (5.4)
Psychiatric conditions	193 (26.2)

Abbreviations: (P25–P75): 25^th^–75^th^ percentiles; SEM, standard error of the mean, n, absolute frequency; n%, relative frequency.

Data presented as medians (P25–P75), means (±SEM) or frequencies [n(n%)].

**Fig. 1:**
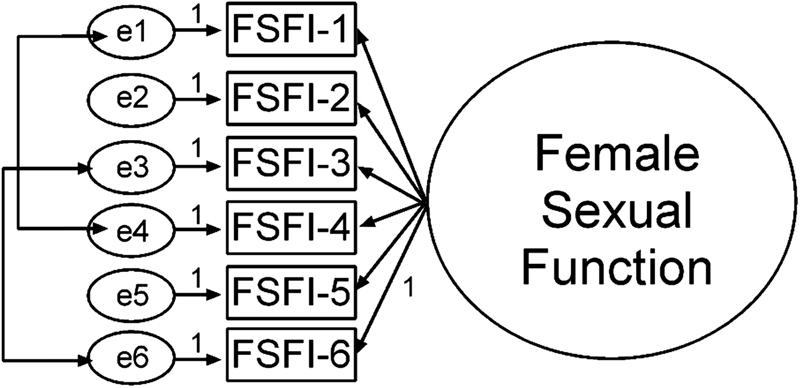
Statistic model—Factor loadings of the variables in each model (AMOS 16.0 software). Abbreviations: FSFI, female sexual function index; e, latent variable. Double arrow in latent variables: correlation between items. FSFI-1 and FSFI-4 correlation: -0.322. FSFI-3 and FSFI-6 correlation: 0.208.

To achieve the cognitive debriefing, 30 climacteric women (*n* = 10 in each menopausal status) were included, and no difficulty was observed in the understanding of the proposed FSFI-6 version (data not shown). The Brazilian version of the questionnaire also underwent an assessment by an expert committee (e.g., three gynecologists, one psychologist, and one biologist). There were no suggestions for changes in this version of the FSFI-6. For the question added at the end of the questionnaire (e.g., “*Is there a word that has not been understood?*”), the answer “yes” was not marked by any of the participants in the validation stage (data not shown). The statistic model used is shown in Fig. 1. Each question of the FSFI-6 was considered a factor loading. The CFA showed an acceptable fit (χ^2^/df = 3.434, CFI = 0.990, TLI = 0.980, RMSEA = 0.058, CI 90% = 0.033–0.083, *p* ≤ 0.001). Good values were evidenced in terms of both factorial weights, as well as regarding the multiple squared correlations (data not shown).

The FSFI-6 and MRS total scores are presented in Table 2, considering menopausal status (pre-, peri-, and postmenopausal) groups. A FSFI-6 total score ≤ 21 is consistent with a positive screening for FSD. The cut-off considered for the Brazilian version is the calculated median of the total scores for the sample (21 points). The frequency of positive screening for FSD, was greater in the postmenopausal group (61.2%) in relation to pre- (43.6%) and perimenopausal (46.6%) women (Chi-Square test, *p* ≤ 0.0001). Peri- and postmenopausal women presented higher menopausal symptoms when compared with the premenopausal group (*p* = 0.001). A good reliability was established in FSFI-6 and MRS (Cronbach α, α = 0.840 and = 0.854, respectively).

**Table 2:** The six-item female sexual function index: Total and each domain scores

Items	Total N = 737	Premenopausal N = 117	Perimenopausal N = 249	Postmenopausal N = 371	*p-value	Cronbach alpha
Desire [minimum–maximum]	3.00 [2.00–3.00] [1.00–5.00]	3.00 [2.00–3.00]^a^ [1.00–5.00]	3.00 [2.00–3.00]^ab^ [1.00–5.00]	3.00 [2.00–3.00]^b^ [1.00–5.00]	**0.015**	0.840
Arousal [minimum–maximum]	3.00 [3.00–4.00] [1.00–5.00]	3.00 [3.00–4.00]^ab^ [1.00–5.00]	3.00 [3.00–4.00]^a^ [1.00–5.00]	3.00 [2.00–3.00]^b^ [1.00–5.00]	**≤** **0.0001**	
Lubrication [minimum–maximum]	4.00 [3.00–4.00] [1.00–5.00]	4.00 [3.00–5.00]^a^ [1.00–5.00]	4.00 [3.00–4.00]^a^ [1.00–5.00]	3.00 [2.00–4.00]^b^ [1.00–5.00]	**0.002**	
Orgasm [minimum–maximum]	4.00 [3.00–4.00] [1.00–5.00]	4.00 [3.00–4.00]^ab^ [1.00–5.00]	4.00 [3.00–5.00]^a^ [1.00–5.00]	3.00 [3.00–4.00]^b^ [1.00–5.00]	**0.010**	
Satisfaction [minimum–maximum]	4.00 [3.00–4.00] [1.00–5.00]	4.00[3.00–4.00] [1.00–5.00]	4.00[3.00–4.00] [1.00–5.00]	4.00 [3.00–4.00] [1.00–5.00]	0.871	
Pain [minimum–maximum]	4.00 [3.00–5.00] [0.00–5.00]	5.00 [4.00–5.00]^a^ [0.00–5.00]	4.00 [3.00–5.00]^ab^ [0.00–5.00]	4.00 [3.00–5.00]^b^ [0.00–5.00]	**0.005**	
Total [minimum–maximum]	21.00 [17.00–24.00] [6.00–30.00]	22.00 [20.11–22.02]^a^ [7.00–30.00]	22.00 [20.29–21.47]^a^ [7.00–30.00]	20.00 [19.17–20.18]^b^ [6.00–30.00]	**≤** **0.0001**	
FSFI total scores ≤ 21	394(53.5)	51(43.6)	116(46.6)	**227(61.2)**	**≤** **0.0001**	
MRS total score [minimum–maximum]	13.00 [7.00–21.00] [0.00–41.00]	12.00 [5.00–17.00]^a^ [0.00–38.00]	13.00 [7.00–22.00]^b^ [0.00–41.00]	14.00 [8.00–21.00]^b^ [0.00–40.00]	**0.001**	0.854

Abbreviations: (P25–P75): 25^th^–75^th^ percentiles; SEM, standard error of the mean; n, absolute frequency; n%, relative frequency; FSFI-6, 6-item female sexual function index; MRS, menopause rating scale; p, statistical significance.

Data presented as medians (P25–P75) or frequencies (n[n%]).

* Kruskal-Wallis (Dunn *posthoc*) and Chi-Square tests with adjusted residual analysis. Bold numbers: association by Chi-Square test with adjusted residual analysis.

ab Different letters indicate statistical significance. Significance set at 5% for all analysis.

The Spearman ρ coefficients between total FSFI-6 scores and variables are displayed in Table 3. Total FSFI-6 scores correlated positively with family income, parity, and educational level, and inversely with age, peri- and postmenopausal status, time of menopause onset, and total MRS score.

**Table 3:** Correlations between the six-item female sexual function index and its variables

Items	FSFI-6 total score (*N* = 737)	
	Coefficient	*p-value
Not having a sexual partner	−0.033	0.375
Single or not living with a partner	0.063	0.087
Female educational level	0.206	≤ 0.0001
Female age	−0.110	0.003
Family income	0.209	≤ 0.0001
BMI	0.038	0.300
Parity	0.097	0.009
Menopause (peri- and postmenopausal status)	−0.154	≤ 0.0001
Time of menopause	−0.107	0.039
Sedentary lifestyle	0.026	0.489
MRS total score	−0.375	≤ 0.0001

Abbreviations: BMI, body mass index (Kg/m2); MRS, menopause rating scale; FSFI-6, 6-item female sexual function index, *p,* statistical significance.

* Spearman correlations. Significance set at 5% for all analysis.

## Discussion

In this study, a Brazilian version of a shorter and faster instrument for FSD screening was translated, culturally adapted and validated. Sexual function and related variables in middle-aged Brazilian women were also assessed.

A CFA and the assessment of the internal consistency were conducted. We found the validity of the FSFI-6 to be satisfactory in a group of 737 women, when compared with previous validation studies around the world.^17^ ^19^ ^20^ ^21^ ^22^ ^33^ The FSFI-6 CFA showed an acceptable fit (χ^2^/df = 3.434, CFI = 0.990, TLI = 0.980, RMSEA = 0.058, CI 90% = 0.033–0.083, *p* ≤ 0.001), and good values were evidenced in terms of both factorial weights, as well as regarding the multiple squared correlations.

Also, this study indicates that the Brazilian FSFI-6 has an excellent reliability (Cronbach α = 0.840) as a screening instrument for FSD in middle-aged women, and the climacteric symptoms and domains measured by MRS reinforced these FSFI-6 properties.

It is important to mention that the present study is the first to provide Brazilian data on the FSFI-6 and MRS. Only women who were sexually active in the last 4 weeks were included. High rates of positive screening for sexual dysfunction were found (53.5%), mostly in postmenopausal women (61.2%). Total FSFI-6 scores were positively correlated with family income, parity, and educational level, and inversely with age, menopause transition (peri- and postmenopausal status), time of menopause onset, and total MRS score.

The menopausal transition defines an impact on the QoL due to biological, social, cultural, physical and psychological aspects, which also affect sexual life.^4^ The FSFI is a well-known tool used to assess the female sexual function, which can be used among pre- and postmenopausal women, in different ethnical populations and medical conditions, with good reliability values.^8^ The FSFI-6 was developed as a shorter and faster alternative, with the same psychometric properties.^17^

Isidori et al^17^ pointed a cut-off value of 19 points for the FSFI-6, which demonstrates a high sensitivity, specificity and positive and negative predictive values for the identification of women with positive screening for FSD in a sample of Italian women. For Lee et al,^22^ the calculated cut-off point for Korean women was 21. Our population differs from the one to which the FSFI-6 was originally designed for in terms of age range, mixed ethnical background as well as different degrees of education and income. In contrast to European^17^ and South Korean^22^ homogeneity, our data was compared with Latin American populations. A large Ecuadorian study used as cut-off point the median calculated from the FSFI-6 scores.^21^ In our study, the same method was used, resulting in a score of ≤ 21 displaying FSD. The computed Cronbach α for the FSFI-6 in this Brazilian research was high, indicating an adequate internal consistency, which was similar to that of the Ecuadorian study.^21^

In this study, 53.5% of the surveyed women displayed scores ≤ 21, suggesting higher risk for sexual dysfunction. This result is comparable to those of the Ecuadorian study, which used the same method for the cut-off value calculation.^21^ Most women with a positive screening for FSD were in the postmenopausal stage (61.2%), similarly to what was observed in previous studies.^21^ ^33^ The worsening sexual function could be explained by postmenopausal status and its consequences, and by aging, once our participants were older than those in the Latin-American studies,^21^ ^33^ and the prevalence of FSD increases with advancing age.^34^ Additionally, Brazilian women had lower to moderate education, multiparity, non-hormone therapy use, and were overweight, a profile characteristic of developing countries.

Although postmenopausal women showed the highest prevalence of positive screening for FSD, perimenopausal women presented 43.6%, suggesting that oscillating levels of estradiol and aging may affect sexual satisfaction.^35^ Besides, brain centers associated with sexual arousal in women, such as amygdale, anterior cingulated cortex (ACC), thalamus, hypothalamus, and insula, seem to exhibit decreased activation in menopause.^36^ These areas are involved in the sexual drive.^36^ Moreover, premenopausal women displayed higher FSFI-6 total scores, hence better sexual function, according to Latin-American studies.^21^ ^33^

The total FSFI-6 scores were positively correlated with family income, parity, and educational level, and inversely with age, peri-, and postmenopausal status, time of menopause onset, and total MRS score. In this research, higher parity was related to better sexual function, in opposition to the literature.^37^ ^38^ The explanation to this discrepancy may rely on the fact that postmenopausal women have independent progeny at this time of life. Besides that, the correlation between parity and sexual function in the peri- and postmenopause had not yet been studied. We speculate that the number of children may confer emotional comfort, but other studies are necessary to verify which variables are associated with parity as a predictive factor of sexual function. Furthermore, high income and educational level can improve female sexual health status possibly due to higher self-care.

Our results agree with other previously published reporting in the literature^8^ ^19^ ^37^ that female aging and postmenopausal status apparently increase the risk for sexual dysfunction. Peri- and postmenopausal women presented higher total symptoms MRS score when compared with the premenopausal group, probably due to progressive estrogenic deficit.^26^ ^29^

As expected, the higher the MRS total score, the worst the female sexual function. The MRS total scores correlated inversely with the FSFI-6 total score, data similar to previously reported researches,^19^ ^37^ demonstrating that multiple factors can contribute to female sexual function and well-being, such as frequency and intensity of menopausal symptoms, social and cultural environment, and other psychological aspects. Besides, the Latin-American society is male-dominated, which can influence the female sexuality.^33^

Therefore, our study confirmed that FSFI-6 is a short and rapid self-reporting instrument and can be combined with other instruments such as the MRS, which also displayed good internal consistencies. Considering these results, the Brazilian version of the FSFI-6 is a consistency tool for assessment of sexual function in middle-aged Brazilian women. In addition, we emphasize that this is a far-reaching study, involving 737 women, which consists in one of the largest populations for FSFI-6 validation up to the present moment.

Certain limitations should be considered. In general, information collected through self-reporting questionnaires lack diagnostic precision.^39^ This may occur due to the difficulty of the subjects understanding the questions, although, as mentioned earlier, this was not the case in the present study (no difficulty was observed in the understanding of the proposed FSFI-6 version). Also, there is a difficulty measuring the extent to which symptoms leads to stress or suffering for each of the subjects. We believe that self-reporting instruments lead to more reliable answers, since the privacy of the participant is preserved when responding. Another important point is the lack of information on the sexual partners of the surveyed women, that should be addressed by a future research, in order do understand this factor involved in multimodal female sexual functioning.

## Conclusion

The FSFI-6 was translated and adapted to the Brazilian culture and is a consistent tool for FSD screening in middle-aged women. Although there is no background data related to sexual function of Brazilian middle-aged women assessed with the FSFI-6, the results of our sample seem to indicate a high frequency of FSD. These results suggest that further investigations about prediction factors are needed, but it already demonstrates the need for more specific instruments for this evaluation in climacteric women.
