# Supplementary material for: Validation of the Six-item Female Sexual Function Index in Middle-Aged Brazilian Women
Source: Rev Bras Ginecol Obstet. 2019 Jul 9;41(7):432–9. doi: 10.1055/s-0039-1692694 (PMC10309272; doi:10.1055/s-0039-1692694)
Supplement: Supplementary file 1 — Supplementary Material [file 10-1055-s-0039-1692694-s180394.pdf]

Supplemental Material 1 The Brazilian 6-item Female Sexual Function Index (FSFI-6)

Nas últimas 4 semanas:						
Como você qualificaria o seu nível (grau) de desejo ou interesse sexual?	Muito alto(5)	Alto(4)	Moderado(3)	Baixo(2)	Muito baixo ou nenhum (1)	
Como você qualificaria o seu nível (grau) de excitação sexual durante a relação sexual ou penetração vaginal?	Não tive atividade sexual nas últimas 4 semanas(0)	Muito alto(5)	Alto(4)	Moderado(3)	Baixo(2)	Muito baixo ou nenhum (1)
Com que frequência você se sentiu lubrificada (notou mais secreção genital) durante a atividade sexual ou penetração vaginal?	Não tive atividade sexual nas últimas 4 semanas(0)	Sempre ou quase sempre (5)	Na maioria das vezes(4)	Às vezes(3)	Poucas vezes (2)	Quase nunca ou nunca(1)
Com que frequência você alcançou o orgasmo quando teve estimulação sexual ou penetração vaginal?	Não tive atividade sexual nas últimas 4 semanas(0)	Sempre ou quase sempre (5)	Na maioria das vezes(4)	Às vezes(3)	Poucas vezes (2)	Quase nunca ou nunca(1)
Quão satisfeita você tem se sentido com a sua atividade sexual?	Muito satisfeita(5)	Moderadamente satisfeita(4)	Nem satisfeita, nem insatisfeita (3)	Moderadamente insatisfeita(2)	Muito insatisfeita(1)	
Com que frequência você sente incômodo ou dor vaginal na penetração?	Não tive penetração vaginal nas últimas 4 semanas(0)	Quase nunca ou nunca(5)	Poucas vezes (4)	Às vezes(3)	Na maioria das vezes(2)	Quase sempre ou sempre(1)

Source: Adapted from Isidori et al.[19] and Pimenta et al.[23]
